# Microarray profile analysis identifies ETS1 as potential biomarker regulated by miR-23b and modulates TCF4 in gastric cancer

**DOI:** 10.1186/s12957-021-02417-w

**Published:** 2021-10-23

**Authors:** Dinglian Mei, Yalong Qi, Yuanyuan Xia, Jun Ma, Hao Hu, Jun Ai, Liqiang Chen, Ning Wu, Daixiang Liao

**Affiliations:** 1 The Department of Oncology, Beijing Mentougou District Hospital, Beijing, 102300 People’s Republic of China; 2 Department of Oncology, Shanghai Pudong New Area Gongli Hospital, Shanghai, 200135 People’s Republic of China

**Keywords:** Gastric carcinoma, miRNAs, Transcription factors, Differentially expressed genes, Bioinformatics analysis, Immunity

## Abstract

**Background:**

Gastric cancer (GC), a common malignancy of the human digestive system, represents the second leading cause of cancer-related deaths worldwide. Early detection of GC has a significant impact on clinical outcomes. The aim of this study was to identify potential GC biomarkers.

**Methods:**

In this study, we conducted a multi-step analysis of expression profiles in GC clinical samples downloaded from TCGA database to identify differentially expressed miRNAs (DEMs) and differentially expressed mRNAs (DEGs). Potential prognostic biomarkers from the available DEMs were then established using the Cox regression method. Gene ontology and Kyoto Encyclopedia of Genes and Genomes (KEGG) enrichment analyses were performed to investigate the biological role of the predicted target genes of the miRNA biomarkers. Then, the prognostic DEM-mediated regulatory network was constructed based on transcription factor (TF)–miRNA–target interaction. Subsequently, the consensus genes were further determined based on the overlap between DEGs and these target genes of DEMs. Besides, expression profile, co-expression analysis, immunity, and prognostic values of these prognostic genes were also investigated to further explore the roles in the mechanism of GC tumorigenesis.

**Results:**

We got five miRNAs, including miR-23b, miR-100, miR-143, miR-145, and miR-409, which are associated with the overall survival of GC patients. Subsequently, enrichment analysis of the target genes of the miRNA biomarkers shown that the GO biological process terms were mainly enriched in mRNA catabolic process, nuclear chromatin, and RNA binding. In addition, the KEGG pathways were significantly enriched in fatty acid metabolism, extracellular matrix (ECM) receptor interaction, and proteoglycans in cancer pathways. The transcriptional regulatory network consisting of 68 TFs, 4 DEMs, and 58 targets was constructed based on the interaction of TFs, miRNAs, and targets. The downstream gene ETS1 of miR-23b and TCF4 regulated by ETS1 were obtained by the regulatory network construction and co-expression analysis. High expression of ETS1 and TCF4 indicated poor prognosis in GC patients, particularly in the advanced stages. The expression of ETS1 and TCF4 was correlated with CD4^+^ T cells, CD8^+^ T cells, and B cells.

**Conclusions:**

miR-23b, ETS1, and TCF4 were identified as the prognostic biomarkers. ETS1 and TCF4 had potential immune function in GC, which provided a theoretical basis for molecular-targeted combined immunotherapy in the future.

## Background

Gastric cancer (GC) is a common malignancy of the human digestive system and represents the second leading cause of cancer-related deaths worldwide [1]. Since early GC is usually mild or asymptomatic, advanced GC is most often diagnosed, leading to difficulties in the diagnosis of GC and poor survival rate of GC patients [2]. Therefore, the identification of specific molecular markers is urgently required in the above processes for clinical applications.

MicroRNAs (miRNAs) are short, approximately 22 nucleotides in length that have no ability of coding proteins and have been suggested to represent transcriptional noise, which widely exists in high eukaryotes [3, 4]. However, more and more evidence indicate that miRNAs play an important role in regulating genes associated with malignant biological behavior in cancer cells [5]. After biogenesis, miRNAs combine the untranslated region (UTR) of the messenger RNAs (mRNAs) of their cognate target genes [6]. MiRNAs as oncogenes (onco-miRNAs) or tumor suppressors (ts-miRNAs) by inhibiting the expression of target genes via cleaving the mRNA molecules or inhibiting their translation, suggests their potential as diagnostic markers of malignancy [7, 8].

Multiple studies have demonstrated the differential expression of miRNAs contributed to malignant phenotypes of GC [9], such as tumor growth, metastasis, angiogenesis, and drug resistance [10, 11]. In fact, they can regulate different signal pathways, targeting genes involved in cell migration, angiogenesis, and cell proliferation [12, 13]. Although molecular characterizations have identified the gene signature for prognosis in GC, today, signatures are still inadequate for accurate patient therapy [14, 15]. Identifying new tumor markers or constructing gene models is still the focus of many studies. Thus, investigating novel biomarkers for early diagnosis and other effective therapies basing on a better understanding of the mechanisms underlying gastric carcinogenesis as well as drug resistance is urgent for improving the outcome of GC patients.

In this study, we conducted a multi-step analysis using various R language packages on clinical samples downloaded from The Cancer Genome Atlas (TCGA) (https://portal.gdc.cancer.gov/) [16] and the Gene Expression Omnibus (GEO) (https://www.ncbi.nlm.nih.gov/geo/) [17] databases to identify DEMs and DEGs. Potential prognostic biomarkers from the available DEMs were then established using the Cox regression method. Subsequently, the target genes of the miRNA biomarkers were predicted by the online analysis tools and multiMIR package in R [18]; and the consensus genes were further determined based on the overlap between DEGs and these target genes. Then, the prognostic DEM-mediated TF–miRNA–target regulatory network was constructed. Furthermore, expression, prognostic values and immunity function of miR-23b downstream genes, ETS1 and TCF4, were explored. Together, the prognostic miRNA and related genes mined in this study will provide new insights in elucidating the molecular mechanisms of gastric cancer and contribute to finding new therapeutic targets and prognostic biomarkers for gastric cancer patients.

## Materials and methods

### Identification of differentially expressed miRNAs and mRNAs

The miRNA and mRNA expression data and the corresponding clinical information from the patients with GC were obtained from the TCGA data portal (https://portal.gdc.cancer.gov/) [16]; a total of 427 samples, including 40 normal and 387 stomach adenocarcinoma (STAD) samples. The differentially expressed miRNAs (DEMs) and mRNAs (DEGs) in normal gastric tissues and GC tissues of TCGA profiles, were calculated using the limma package with the voom method in R, separately [19, 20]. Adjusted *P* value < 0.05 and |log2 fold change (FC)| > 1 were set as cutoff value. The results were visualized with the ggplot2 package in R [21].

### Survival analysis of DEMs

The DEM profiles were normalized by log2 transformation. Expression of the DEMs associated with overall survival (OS) in GC patients were analyzed by univariate Cox regression. Variables with *P* value < 0.05 in univariate Cox regression were further used for multivariate Cox regression analysis to determine whether they could function as independent prognostic factors along with the clinical factors (including age; gender; and clinical stage, lymph nodes, and distant metastasis). The hazard ratios (HRs) with 95% confidence intervals (CI) and log-rank *P* values were also computed. The results were visualized with the “forestplot” package of R. Survival curves of the genes were evaluated and plotted using the Kaplan-Meier method and the log-rank test by the “survival” package in R (https://CRAN.R-project.org/package=survival) [22]. The log-rank test was used to evaluate statistical significance with a cutoff criterion of *P* < 0.05 [23].

### GO terms and KEGG pathway enrichment analysis of DEMs and targets

The target genes of prognostic DEMs were predicted using TargetScan (http://www.targetscan.org/), TarBase (http://www.mirdb.org/miRDB/), and DIANA-microT (http://www.microrna.gr/webServer) online analysis tools and multiMIR package in R [24]. To further enhance the bioinformatics analysis reliability, the overlapping target genes were obtained using the Venn diagram. Then, the overlapped genes were analyzed by The Database for Annotation, Visualization, and Integrated Discovery (DAVID) bioinformatics tool (https://david.ncifcrf.gov/) [25]. Gene Ontology (GO) and Kyoto Encyclopedia of Genes and Genomes (KEGG) pathway enrichment analyses were then performed for the target genes. The *P* value < 0.01 was set as the cutoff criterion [26, 27]. Furthermore, the targets of the transcription factor were predicted using TRANSFAC (http://gene-regulation.com/) and Harmonizome (http://amp.pharm.mssm.edu/Harmonizome/) online databases [28, 29].

### Construction of TF–DEM–target gene regulatory network

Interactions of DEMs and target genes were obtained from the reliable online miRNA–mRNA databases, including miRDB, TargetScan, miRTarBase, etc., using the “multiMiR” package in R (http://multimir.org/) based on the experimental verification of luciferase reporter assay. To identify the transcription factors (TFs) that are targeted miRNAs, we used published TransmiR databases [22]. Then miRNAs both in miRNA–target interaction and TF–miRNA interaction were obtained. Consequently, the integrated dysregulated TF–DEM–target regulatory network was constructed and visualized using Cytoscape software.

### Expression validation of DEMs and targets

The expressions of DEMs and targets were further analyzed with the TCGA dataset. The Gene Expression Profiling Interactive Analysis (GEPIA2) (http://gepia2.cancer-pku.cn/#index) and UALCAN (http://ualcan.path.uab.edu/index.html) databases, which are the publicly accessible online cancer databases integrating sequencing data from several database, were used to validate expressions of DEMs and targets in different statuses. The mRNA expression was analyzed using the unpaired Student’s *t* test. The expression value was considered statistically significant when the *P* value < 0.05.

### Correlation analysis of has-mir-23b target, ETS1

The LinkedOmics database (http://www.linkedomics.org/login.php) was used to explore the ETS1 co-expression genes by using Pearson’s correlation coefficient and showed the results with volcano plot and heatmaps. Furthermore, the KEGG pathways of ETS1 and its co-expression genes were explored by using gene set enrichment analysis (GSEA).

### Correlation and expression of ETS1 and its target TCF4 in TILs of STAD

The correlation between ETS1 and its target TCF4 expression and the major tumor infiltrating lymphocytes (TILs), including B cell, CD4^+^ T cell, CD8^+^ T cell, etc., was explored via the TIMER database (https://cistrome.shinyapps.io/timer/), which collected 32 cancer types from TCGA. The *P* value < 0.05 was statistically significant. Then, the expression of ETS1 and its target TCF4 in TILs between tumor and normal tissues were analyzed in GEPIA2021 database (http://gepia2021.cancer-pku.cn/).

### Statistical analysis

All analyses were conducted using RStudio software (version 1.3.959). Student's *t* test was conducted to compare the differences. And a two-sided *P* < 0.05 was regarded as significant.

## Results

### Identification of five DEMs associated with OS in STAD

**Fig. 1:**
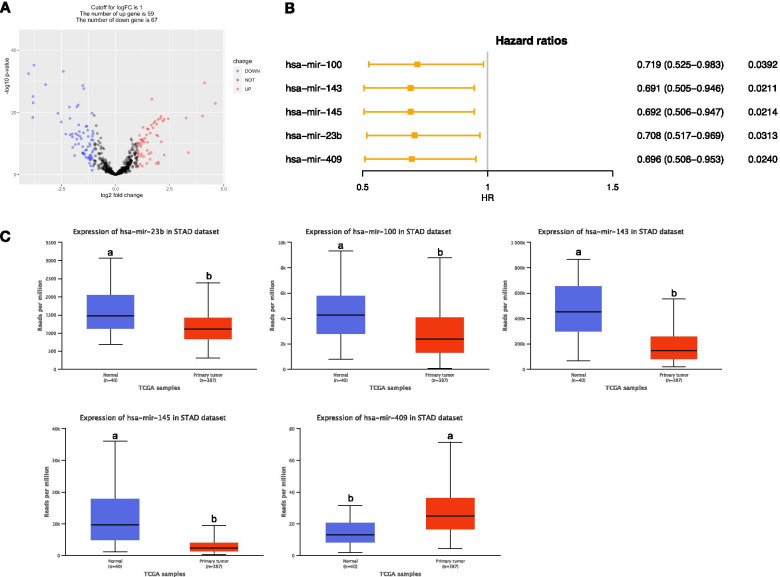
Five DEMs associated with overall survival in STAD patients. **A** Volcano plot of DEMs between STAD and normal tissues. The red dot represents upregulated miRNAs, and blue dot represents downregulated miRNAs. **B** Forest plot of prognostic values of miRNAs based on the Cox regression analysis. **C** The boxplots of expression levels of DEMs between STAD and normal tissues. Different letters upon the box plot illustrated significant expression difference (*P* < 0.001)

A total of 126 DEMs were obtained after analyzing miRNA expression profiles from TCGA with R language using adjusted *P* value < 0.05 and |log2FC| > 1 as screening criteria. Among them, 67 miRNAs were significantly downregulated, and 59 miRNAs were significantly upregulated. The volcano map illustrates the significant differences and distribution of the fold change in DEMs (Fig. 1A). Five prognostic associated DEMs were obtained through the univariate and multivariate Cox regression analysis. Multivariate Cox regression analysis revealed that mir-100 (*HR* = 0.719, *P* < 0.05), mir-143 (*HR* = 0.691, *P* < 0.05), mir-145 (*HR* = 0.692, *P* < 0.05), mir-23b (*HR* = 0.708, *P* < 0.05), and mir-409 (*HR* = 0.643, *P* < 0.05) had negative coefficients, indicating that the lower expression level of these DEMs were associated with better patients’ OS (Fig. 1B). In addition, except mir-409, the other four DEMs were significantly downregulated, in STAD tissues compared with normal tissue (*P* < 0.001, Fig. 1C).

### Prediction and enrichment analysis of prognostic targets of DEMs

**Fig. 2:**
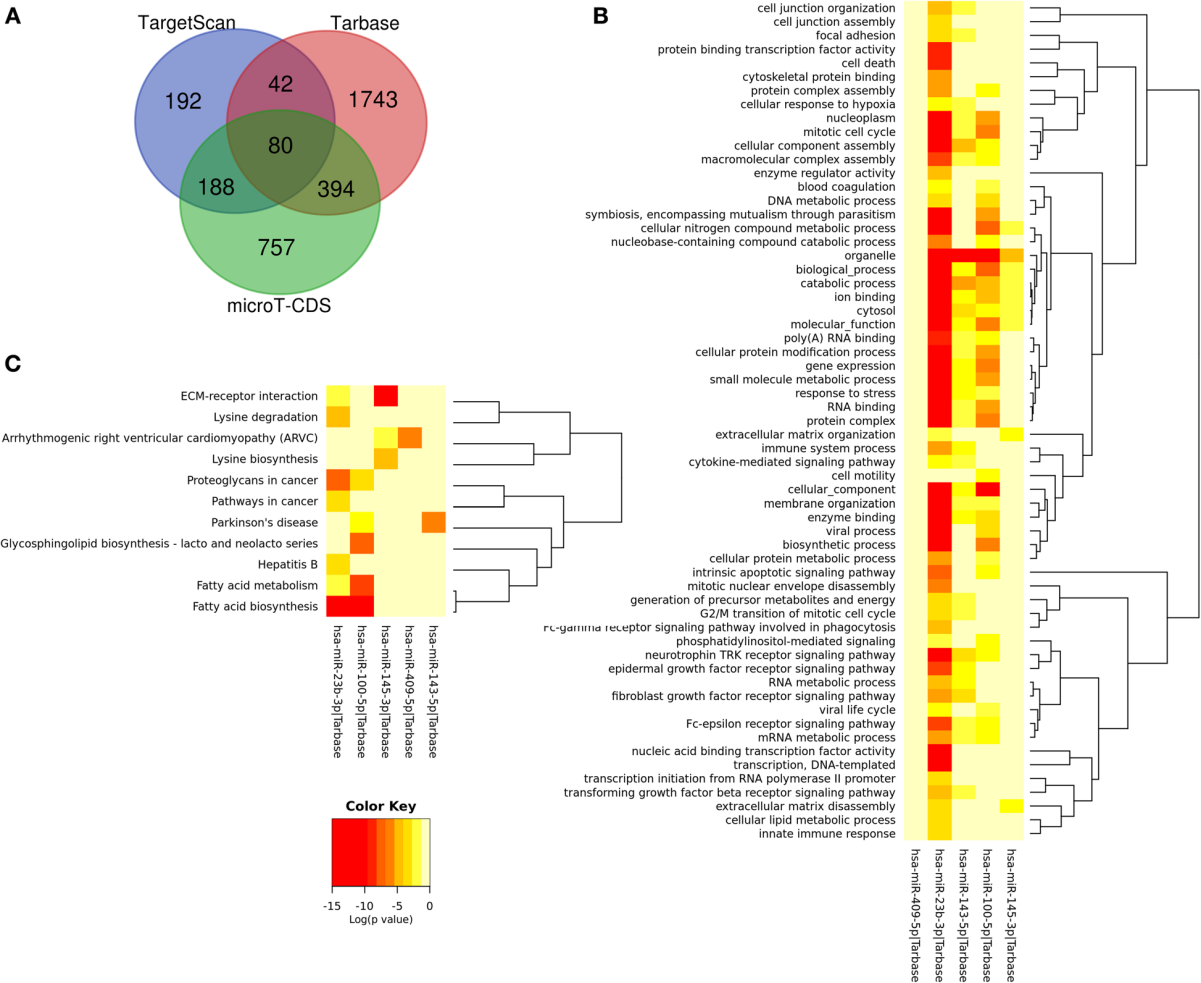
Heatmaps of enrichment analysis of the targets of five prognostic DEMs. **A** Venn diagrams for the targets of five prognostic DEMs using different analysis tools; 80 genes were overlapped. **B** The significant enriched GO terms of target genes. **C** The significant enriched KEGG pathways of target genes

According to the DEM–target gene analysis, we got 3396 candidate genes of the five prognostic DEMs. A Venn diagram was generated for the comparison of the target gene numbers of DEMs using different analysis tools; thus, 80 common targets of DEMs were acquired (Fig. 2A). Furthermore, enrichment analysis of target genes of single miRNA was also performed. As shown in the heatmap, among five DEMs, targets of miR-23b and miR-100 were significantly enriched in most GO terms and KEGG pathways. The common GO terms of miR-23b, miR-100, and miR-143 targets were enriched in organelle and catabolic process (Fig. 2B). Additionally, targets of miR-23b and miR-100 were enriched in fatty acid metabolism and biosynthesis pathway. Meanwhile, targets of miR-143, miR-409, and miR-145 were enriched in Parkinson’s disease, arrhythmogenic right ventricular cardiomyopathy, and ECM–receptor interaction, respectively (Fig. 2C).

### TF–miRNA–target transcriptional regulatory network

**Fig. 3:**
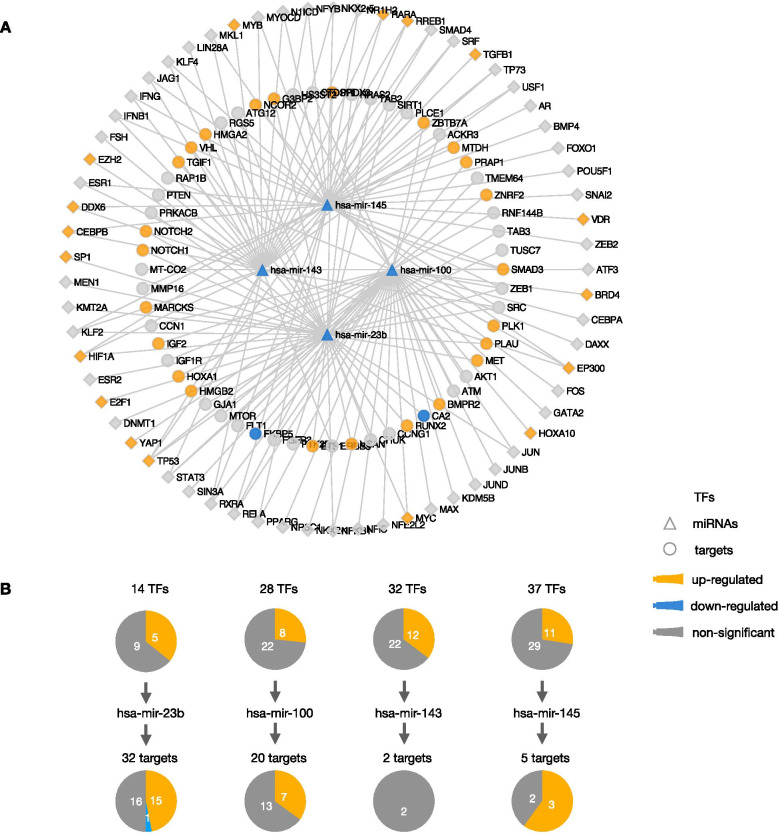
TF–miRNA–target network associated with the five prognostic DEMs. **A** The TF–miRNA–target network; **B** the differentially expressed TFs and targets related to each DEM

To better understand the role of identified prognostic DEMs, we further constructed a transcriptional regulatory network based on the interaction of TF, miRNA, and targets. First, 68 TFs targeting 4 prognostic DEMs except mir-409 were identified. At the same time, 74 targets verified by luciferase reporter experiment interacted with 5 prognostic DEMs by using R “multiMiR” software package. After unifying the interactions between TFs and DEMs and between DEMs and targets, we constructed a transcriptional regulatory network consisting of 68 TFs, 4 DEMs, and 58 targets (Fig. 3A). Next, we analyzed the expression levels of TFs and targets in the transcriptional regulatory network. The results showed that there were 18 upregulated TFs, 24 targets, and 2 downregulated targets in gastric cancer. The single DEM analysis showed that there were the most differentially expressed target genes in the regulatory pathway of mir-23b, including 15 upregulated targets and one downregulated target gene (Fig. 3B). Further analysis showed that among the upregulated targets, 5 were TFs. The results revealed that the downstream of mir-23b regulate more gene expression and pathways, which indicates that mir-23b plays an important role in the occurrence and development of gastric cancer.

### Downstream genes regulated by mir-23b

**Fig. 4:**
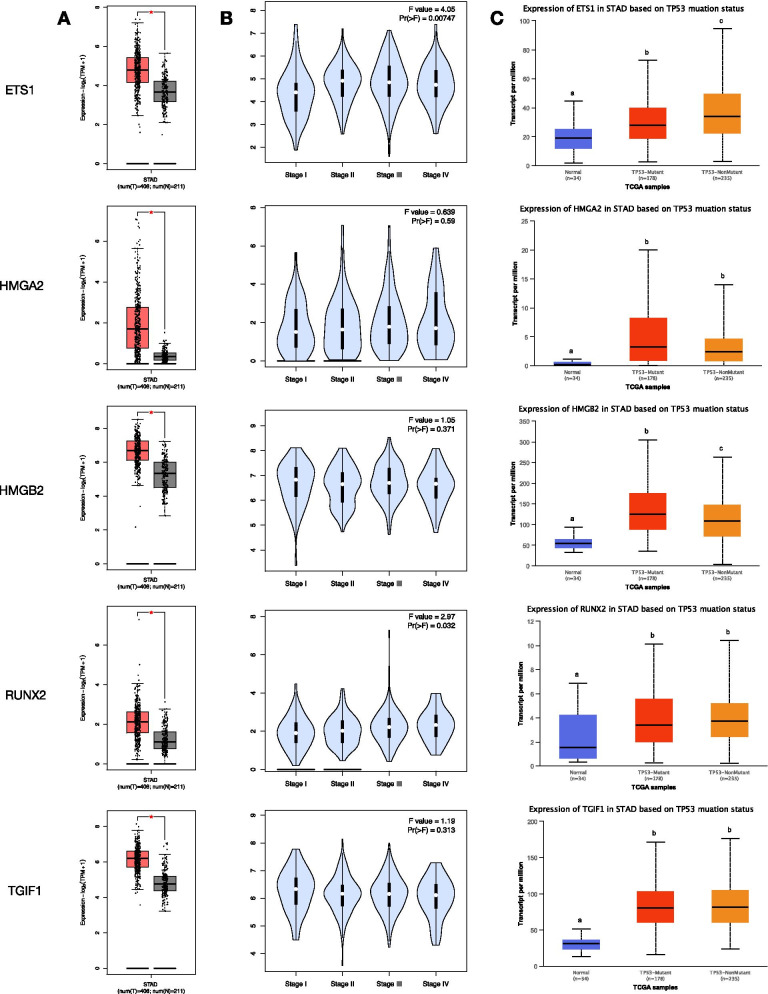
Expression patterns of five targets associated with mir-23b. **A** The boxplots of expression levels of five targets between GC and normal tissues in TCGA dataset. ^*^P < 0.05. **B** The violin plots indicated the expression levels of five targets in stages I–IV of GC. **C** The boxplots of expression levels of five targets based on TP53 mutant status. Different letters upon the box plot illustrated a significant expression difference (*P* < 0.001)

Compared with the normal tissues in the TCGA dataset, the expression of 5 TFs regulated by mir-23b was upregulated in STAD tissue samples (*P* < 0.05, Fig. 4A). In addition, with STAD development, the expression level of ETS1 was significantly upregulated in advanced stages (stages II, III, and IV), and the expression level of RUNX2 was significantly upregulated in stages III and IV (*P* < 0.05, Fig. 4B). Further analysis revealed that the expression of ETS1 was also affected by the TP53 mutation. In contrast to normal tissues, the expression level of ETS1 in STAD samples with TP53 mutation was significantly upregulated, while the expression level of ETS1 in STAD samples with TP53 nonmutation was further significantly upregulated (*P* < 0.001, Fig. 4C).

### Correlation analysis of mir-23b target ETS1

**Fig. 5:**
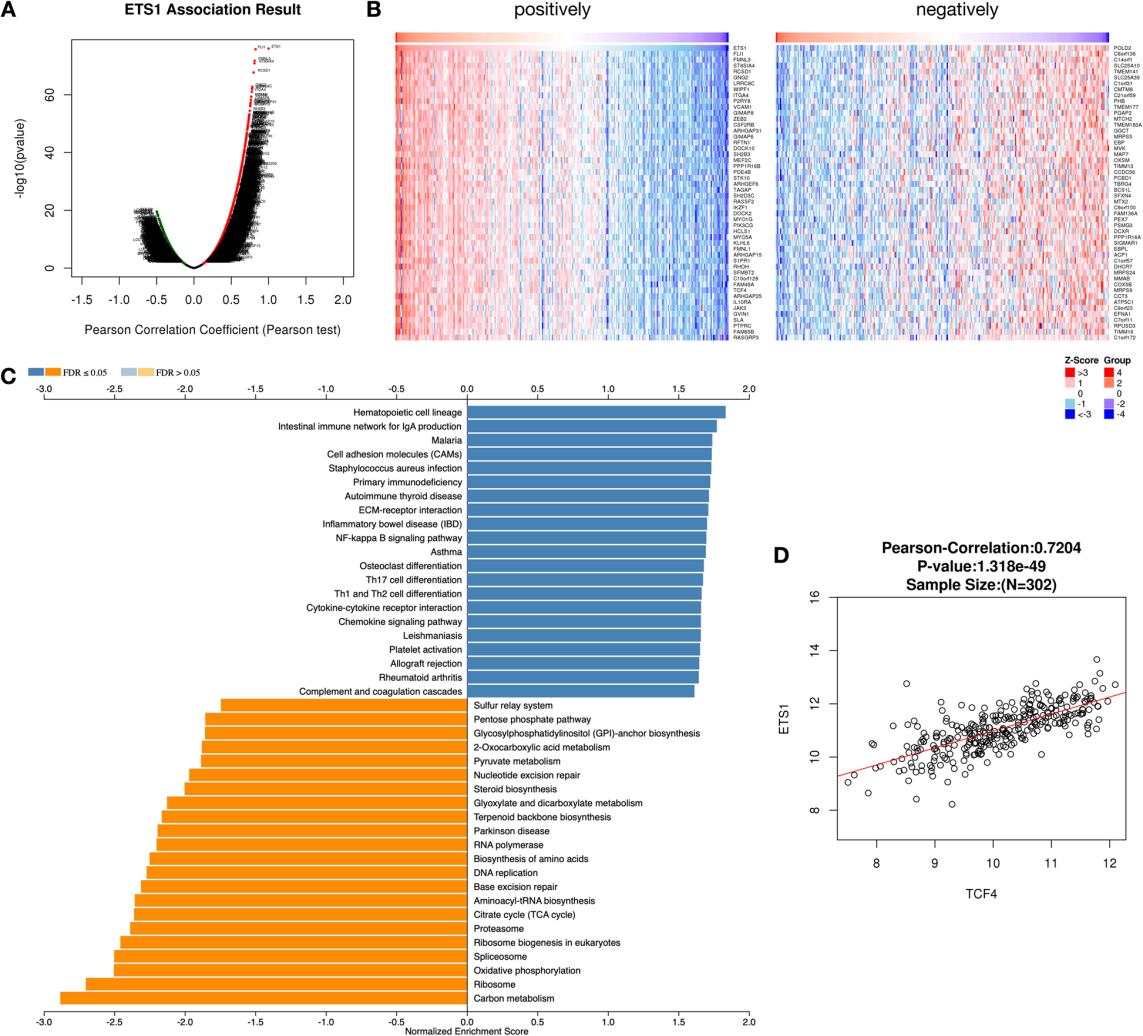
Correlation expression genes of mir-23b target gene ETS1. **A** The volcano plot indicated the Pearson correlation coefficient between ETS1 and related genes. **B** The heatmaps indicated the positively and negatively correlated genes associated with ETS1, respectively. **C** The KEGG pathways related to the correlated gene to ETS1 based on GSEA analysis. **D** The Pearson correlation coefficient between ETS1 and downstream gene TCF4

Next, we analyzed the co-expressed genes of ETS1 and found that 1771 positively correlated genes (*r* > 0.4, *FDR* < 0.01) and 130 negatively correlated genes (*r* < − 0.4, *FDR* < 0.01) (Fig. 5A, B). Later, to explore the biological pathways that co-expressed genes of ETS1 may participate in, we performed KEGG pathway enrichment analysis on these co-expressed genes based on the GSEA method. The results showed that ETS1 co-expressed genes are widely involved in various cellular development pathways. We found that the ETS1 co-expressed genes were positively enriched in the immune-related pathways, including primary immunodeficiency, autoimmune thyroid disease, Th1 and Th2 cell differentiation, chemokine signaling pathway, etc.. At the same time, the ETS1 co-expressed genes were mainly positively enriched in sulfur relay system, steroid biosynthesis, RNA polymerase, DNA replication, etc. (Fig. 5C).

### Correlation and expression of ETS1 and its target TCF4 in TILs of STAD

**Fig. 6:**
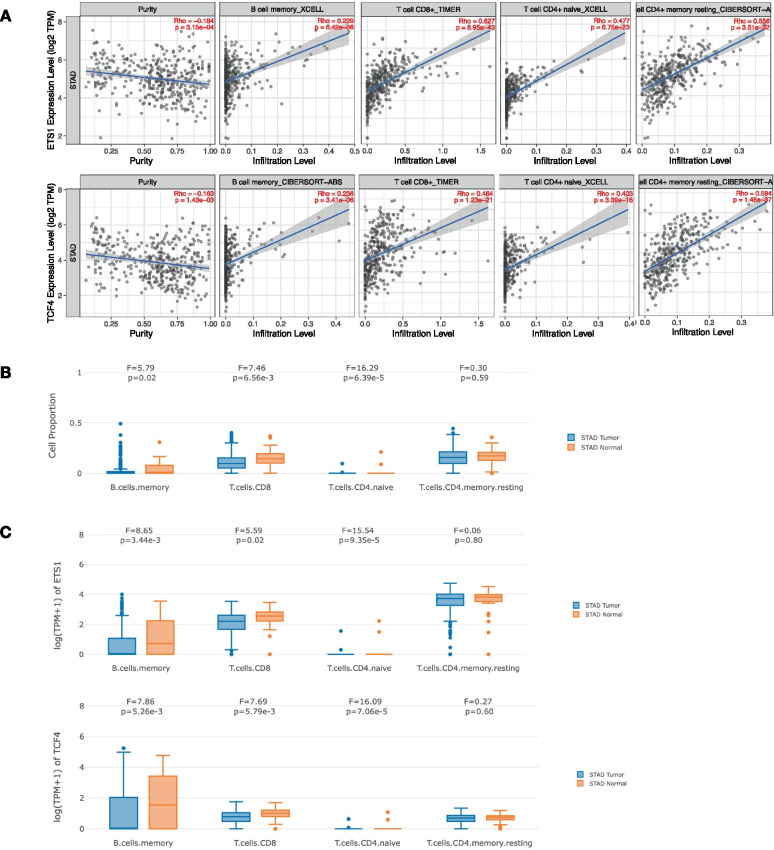
The relationship between ETS1 and downstream gene TCF4 expression and abundance of immune cells. **A** The relationship between ETS1 and downstream gene TCF4 expression and abundance of B memory cell, CD8^+^ T cell, CD4^+^ T cell, and CD4^+^ memory resting T cell. **B** The cell proportion in immune infiltrates between STAD tumor and normal tissue. **C** The expression box plots of ETS1 and TCF4 between tumor and normal tissue in immune infiltrates in STAD, respectively

Among the co-expressed genes of ETS1, we found that the expression level of TCF4, a downstream gene of ETS1, was positively correlated with that of ETS1 (*r* = 0.7204, *P* = 1.318e–49, Fig. 5D). Next, we analyzed the relationship between ETS1 and TCF4 and the abundance of immune cells. The results showed that the expression of ETS1 and TCF4 in STAD tissues was positively correlated with the abundance of B cells, CD8^+^ T cells, and CD4^+^ T cells and negatively correlated with the abundance of CD4^+^ memory resting T cells and CD4^+^ Th1 T cells (Fig. 6A). Next, we examined the difference in cell proportion of TILs in STAD tissue and normal tissue. The results showed that the cell proportion of B memory cell (*P* = 0.02), CD8^+^ T cell (*P* = 6.56E–3) and CD4^+^ naive cell (*P* = 6.39E–5) in STAD tissues was significantly lower than that in normal tissues (Fig. 6B). The expression levels of ETS1 and TCF4 genes in these three immune cells were also significantly higher in normal tissues than in STAD tissues (Fig. 6C).

### Prognostic values of ETS1 and TCF4 in STAD

**Fig. 7:**
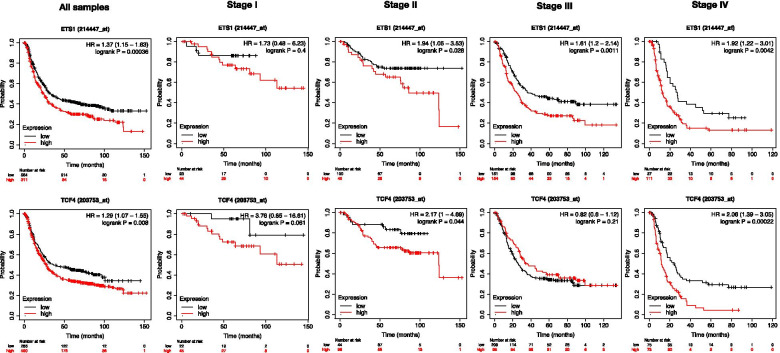
Kaplan-Meier curve and log-rank test of TCGA samples categorized by the expression of ETS1 and TCF4. The patients were stratified into high-level group and low-level group according to the median of each gene

To investigate the prognostic significance of ETS1 and TCF4, the log-rank test and Kaplan-Meier method were performed to investigate the relationship of the expression and OS of STAD patients. Subsequently, the results revealed ETS1 (*HR* = 1.37, *P* < 0.001) and TCF4 (*HR* = 1.29, *P* = 0.008) were found to be closely related to OS in STAD patients. The results indicated that the lower expression levels of ETS1 and TCF4 were associated with better patients’ OS (Fig. 7). As the expression analysis results showed that the ETS1 expression level increased significantly along with the STAD development, we analyzed the correlations between the expression of ETS1 and TCF4 and OS in STAD patients with advanced stages. The results showed that compared with no significant correlation between the patients’OS with stage I and the expression of ETS1 and TCF4, patients with stages II to IV and low expression of ETS1 or patients with stages II and IV and low expression of TCF4 had significantly better OS (*P* < 0.05, Fig. 7).

## Discussion

In the present study, we got expression profiles of miRNAs and mRNAs in STAD and normal tissues from the TCGA dataset. Then, we identified 126 significantly differentially expressed miRNAs, including miR-100, miR-23b, miR-143, miR-145, and miR-409, which were also associated with the overall survival in STAD patients. Enrichment analysis showed that the GO terms were mainly enriched in nuclear chromatin, organelle, RNA binding, cellular protein modification process, mRNA catabolic process, etc.. The KEGG pathways were significantly enriched in cancers, cellular community, infectious disease, signal transduction, and cellular senescence. Among these five DEMs, targets of miR-23b and miR-100 were significantly enriched in the same GO terms and KEGG pathways, which suggested that they may have similar functions during STAD progression. To better understand the role of identified prognostic DEMs, a transcriptional regulatory network consisting of 68 TFs, 4 DEMs, and 58 targets was constructed based on the interaction of TFs, miRNAs, and targets. The regulatory axis of the single DEM analysis showed 16 differentially expressed target genes in the regulatory pathway of mir-23b, including 5 upregulated TFs. The results suggested that more gene expression and pathways are regulated by miR-23b, which indicates that mir-23b plays an important role in the occurrence and development of gastric cancer. Among these 5 upregulated TFs which were downstreams of miR-23b, ETS1 was significantly upregulated in advanced stages and differentially affected by TP53 mutation. Next, co-expression genes of ETS1 were obtained and positively enriched in the immune-related pathways, indicating that ETS1 has a potential immune function in STAD progression. Furthermore, it was verified by the analysis of the correlation and expression of ETS1 and its highly correlated target TCF4 in TILs in STAD.

miR-23b, belonging to the miR-23b~27b~24-1 cluster (9q22.32), is a pleiotropic modulator in different organs especially associated with cancer development [30, 31]. Transcription factors TP53 and p65 (a subunit of the NFkB protein) upregulate gene expression and dimerization of Jun and Fos (AP-1) and repress the transcription of the pre-miRNA cluster [32]. The findings on miR-23b indicate that it is a very potent post-transcriptional regulator of growth and differentiation during development, multiple cancers, and other biological processes [33, 34]. In the present study, miR-23b was found downregulated in STAD tissues suggesting that it acted as a tumor suppressor. Furthermore, the expression levels of upstream transcription factors MYC, TP53, E2F1, HIF1A, and SP1 in miR-23b were upregulated, indicating the negative regulation of these transcription factors on miR-23b. Because these transcription factors are involved in different cellular pathways, it is concluded that miR-23b may regulate the occurrence and development of gastric cancer in many ways under different conditions. This result can be confirmed by other cancer studies. c-Myc is upregulated in prostate cancer cells and inhibits miR-23a and miR-23b at the transcriptional level, resulting in its target protein mitochondrial glutaminase overexpression [35]. Using miRNA microarray meta-analysis, it was found that miR-23b was associated with hypoxia-related pathways mediated by the HIF gene family, which revealed the important role of miR-23b in hypoxia and thrombosis pathway [36]. E2F1 binding sequence participates in the expression of C9orf3 transcripts of host genes in miR-23b/27b/24 clusters, thus promoting cell migration [37]. The expression of miR-23b is downregulated in multiple myeloma (MM) and Waldendtrom's macroglobulinemia (WM) cells, and promoter methylation is one of the mechanisms of miR-23b inhibition [38]. However, we found that there was no differential expression in the upstream DNMT1 of miR-23b in our study. It is speculated that the expression of miR-23b in gastric cancer may not be related to methylation.

**Fig. 8:**
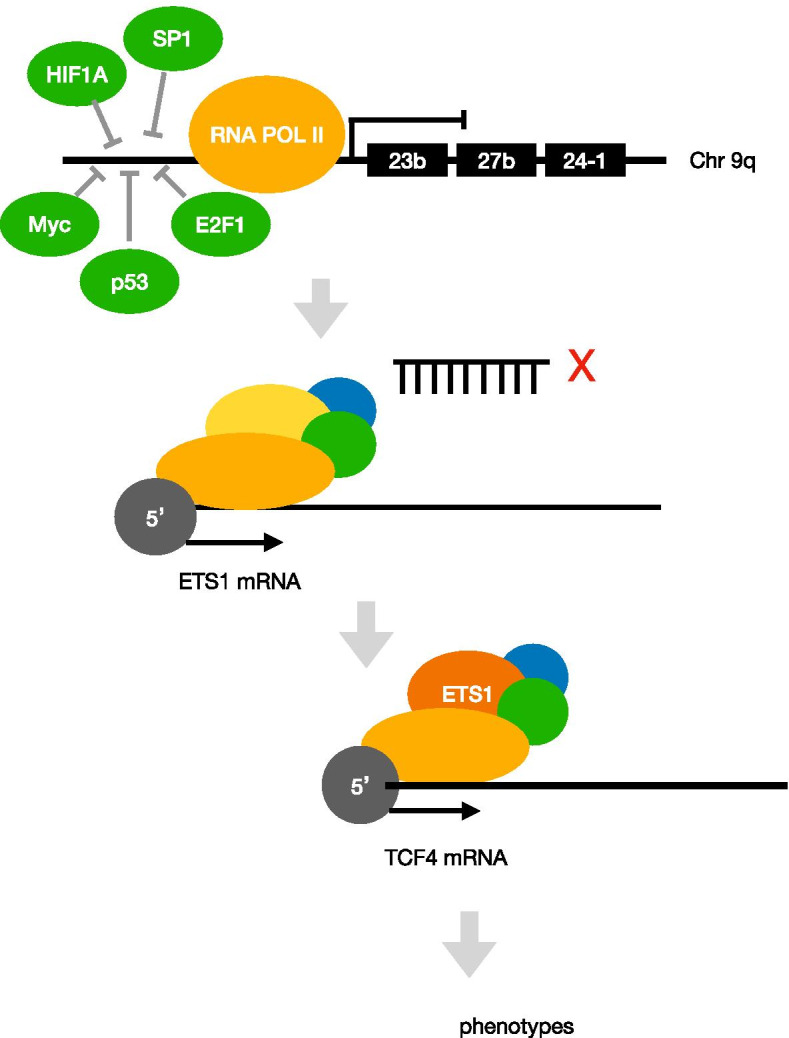
Transcriptional model of miRNA23b regulated by TFs and effecting the downstream genes ETS1 and TCF4

The downstream gene ETS1 of miR-23b and TCF4 regulated by ETS1 were obtained by the regulatory network construction and co-expression analysis. ETS1 is a kind of oncogene, which is frequently upregulated in human tumors from different tissue sources. ETS1 can inhibit cell differentiation in many different situations and promote its cancer-promoting effect by keeping cells in a state of immaturity and proliferation [39]. It was found that ETS1 can directly induce the expression of key transcription factors (E4BP4, TXNIP, TBET, GATA3, HOBIT, BLIMP1) that regulate the differentiation of NK cells, and regulate the expression of apoptosis and activation-related genes in NK cells [40]. In the process of thymus CD8 T cell differentiation, ETS1 plays an important role in the inhibition of CD4 and the upregulation of Runx3 [41]. β-catenin is the molecular hub of the Wnt signaling pathway, and TCF4 is a transcription factor of the Wnt pathway, which is highly expressed in colorectal cancer [42, 43]. WNT/β-catenin signal is involved in many physiological processes and pathological events, including embryonic development, cell migration and polarization, maintenance and proliferation of cancer stem cells (CSC), and epithelial–mesenchymal transformation [44, 45]. In addition, β-catenin/TCF pathway promotes tumor immune tolerance in DCs, RA, and Tregs and plays an important role in tumor immunotherapy. For a long time, the Wnt/β-catenin pathway represented by CTNNB1, LEF1, TCF4, etc. has been considered to regulate EMT, and ETS1 is also involved in epithelial–mesenchymal transition (EMT) [46]. Studies have found that miR-155 inhibits the EMT of cells by targeting TCF4, a key regulator of EMT [47]. As a well-recognized cancer driver gene, β-catenin has been demonstrated as a molecular target for developing anticancer drugs. Many natural products and small-molecule inhibitors have been discovered to inhibit the β-catenin/TCF binding or promoting β-catenin phosphorylation and ubiquitination, including fisetin [48], parthenolide [49], γ-mangostin [50], curcumin [51], etc.. These inhibitors can also be used in the treatment of patients with gastric cancer. Our study found that mir-23b affects the expression of TCF4 by regulating ETS1, and mir-23b may be negatively regulated by several upstream transcription factors (Fig. 8). Furthermore, high expression of ETS1 and TCF4 indicated poor prognosis, suggesting that ETS1 and TCF4 are potential prognostic biomarkers for gastric cancer. In fact, ETS1 and TCF4 expression are significantly upregulated in advanced gastric cancer tissues, suggesting that ETS1 and TCF4 may be involved in tumor growth and progression. In our analysis of the ETS1 co-expression network, we found that ETS1 and its co-expressed genes were indeed involved in regulating the immune response. The expression of ETS1 and TCF4 was correlated with CD4^+^ T cells, CD8^+^ T cells, and B cells, suggesting that ETS1 and TCF4 had potential immune function in STAD. There was a strong correlation between ETS1 and TCF4, which provided a theoretical basis for molecular targeted combined immunotherapy in the future. Taken together, these results strongly suggested the potential of ETS1 and TCF4 as targets in anticancer immunotherapy.

However, despite our comprehensive and systematic analysis of ETS1 and TCF4 and cross-validation using different databases and R 3.2.2, there are some limitations to this study. The mechanism of ETS1 and TCF4 synergistic involvement in immune regulation is still unclear, and the specific pathway needs further study. In the future, prospective studies of ETS1 and TCF4 expression and their role in immune invasion of human cancers are needed, and a novel antitumor immunotherapeutic agent targeting ETS1 and TCF4 is successfully developed and tested.

## Conclusion

In conclusion, we systematically identified DEMs and DEGs through TCGA datasets. We got five miRNAs, including miR-23b, miR-100, miR-143, miR-145, and miR-409, which are associated with the overall survival of GC patients. Subsequently, pathway and GO enrichment analysis of targets of DEMs gave us an insightful view of the functions of DEMs. Then, the transcription factor ETS1 was further determined through the TF–miRNA–target regulatory network. Furthermore, TCF4, which is the downstream genes of ETS1, was also upregulated in STAD tissues. Together, miR-23b, ETS1, and TCF4 were identified as prognostic biomarkers. ETS1 and TCF4 had potential immune function in STAD, which provided a theoretical basis for molecular targeted combined immunotherapy in the future.

## Data Availability

The datasets used during the present study are available from the corresponding author upon reasonable request.

## Abbreviations

DAVID: The Database for Annotation Visualization and Integrated Discovery
DEGs: Differentially expressed mRNAs
DEMs: Differentially expressed miRNAs
EMT: Epithelial-to-mesenchymal transition
FC: Fold change
GEO: The Gene Expression Omnibus
GC: Gastric cancer
GO: Gene Ontology
KEGG: Kyoto Encyclopedia of Genes and Genomes
miRNAs: MicroRNAs
mRNAs: Messenger RNAs
K-M: Kaplan–Meier
OS: Overall survival
STAD: Stomach adenocarcinoma
TCF4: Transcription factor 4
TCGA: The Cancer Genome Atlas
